# Long‐term treatment outcomes in gastric cancer with oligometastasis

**DOI:** 10.1002/ags3.12733

**Published:** 2023-08-31

**Authors:** Kentaro Hara, Haruhiko Cho, Atsushi Onodera, Kazuya Endo, Yukio Maezawa, Toru Aoyama, Takanobu Yamada, Takashi Oshima, Yasushi Rino

**Affiliations:** ^1^ Department of Gastric Surgery Tokyo Metropolitan Cancer and Infectious Diseases Center Komagome Hospital Tokyo Japan; ^2^ Department of Surgery Yokohama City University Yokohama Japan; ^3^ Department of Gastrointestinal Surgery Kanagawa Cancer Center Yokohama Japan

**Keywords:** chemotherapy, gastrectomy, gastric cancer, metastasectomy, oligometastasis

## Abstract

**Aim:**

While surgery is essential for curative treatment of gastric cancer with oligometastasis, its target, timing, and possibility of combination with other treatments are unclear. We herein investigated the clinical course and long‐term outcomes of gastric cancer with oligometastasis in the real world setting to determine the optimal therapeutic strategy.

**Methods:**

The present study retrospectively analyzed 992 patients who received any treatment for metastatic or recurrent gastric adenocarcinoma at Tokyo Metropolitan Komagome Hospital between 2007 and 2019. Oligometastasis was defined as any one of the following: liver metastases (HEP) <3; lung metastases (PUL) <3; unilateral adrenal gland metastasis (ADR); para‐aortic lymph node metastasis (PALN); or one, distant, lymph node metastasis, excluding the regional lymph nodes (LYM). Overall survival was compared by the characteristics and treatments for the oligometastasis, and univariate and multivariate analyses were used to identify the prognostic factors of overall survival.

**Results:**

Ninety‐seven patients (9.8%) with the following metastasis sites were enrolled: HEP (*n* = 27), PUL (*n* = 2), ADR (*n* = 3), PALN (*n* = 55), and LYM (*n* = 10). The median survival time of the cohort was 22.8 months, and the five‐year overall survival rate was 28.4%. On multivariate analysis, chemotherapy for the initial treatment (hazard ratio [HR]: 0.438; *p* = 0.048), distal gastrectomy and/or metastasectomy (HR: 0.290; *p* = 0.001), and R0 resection (HR: 0.373; *p* = 0.005) were identified as independent, positive factors of overall survival.

**Conclusion:**

The long‐term outcomes of gastric cancer in patients with oligometastasis may improve if treatment is begun with chemotherapy rather than surgery.

## Graphical abstract

The present study investigated he clinical course and long‐term outcomes of 97 gastric cancer patients with oligometastasis. The patients with oligometastasis had a favorable prognosis, with a median survival time of 22.8 months and a 5‐year overall survival rate of 28.4%. On multivariate analysis, chemotherapy for the initial treatment, distal gastrectomy and/or metastasectomy, and R0 resection were identified as independent, positive factors of overall survival.

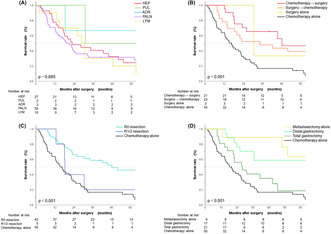

## 1 INTRODUCTION

Gastric cancer is the third most‐common cancer and the second leading cause of cancer‐related deaths in the world.^1^ While surgical intervention is essential for achieving a cure for locally advanced gastric cancer, chemotherapy is the standard of care for stage IV gastric cancer with a distant metastasis because of its non‐curability by local therapy. Oligometastasis is roughly defined as a condition having a small number of metastases distant from the primary tumor. Its curability has been much discussed owing to the challenges posed by its local and systemic aspects that depend on the biological characteristics of the primary organ affected.^2^ Oligometastasis in gastric cancer is relatively rare, occurring only occasionally in the lungs, liver, and non‐regional lymph nodes. Although prospective studies of this form of cancer are scarce because of the small number of cases and the variety of metastatic sites, several case series demonstrating a survival benefit of surgical intervention have been published.^3^, ^4^, ^5^, ^6^, ^7^ Based on the accumulation of such data, the 6th edition of the Japanese gastric cancer treatment guidelines, published in 2021, provide a weak recommendation for surgery as means of improving long‐term outcomes in patients with oligometastasis, especially of the para‐aortic lymph node at station #16 a2/b1 or a single liver metastasis.^8^

However, such recommendations are subdivided into organ‐specific ones because of the different levels of invasiveness and difficulties with surgery related to the anatomical location of the tumor and therefore cannot be generalized to other cases until comprehensive, organ‐independent evidence for the treatment of oligometastasis of gastric cancer is established. Moreover, although previous studies in Europe reported that a combination of local and systemic therapy had the potential to improve long‐term survival in patients with gastric cancer with oligometastasis,^9^, ^10^ it remains unclear whether surgery or chemotherapy should be the initial treatment. As for postoperative chemotherapy, previous clinical trials found that patients who underwent a gastrectomy had poor tolerance for intensive chemotherapy with highly toxic agents, such as platinum.^11^, ^12^, ^13^ The REGATTA trial,^13^ an international, open‐label, randomized, phase‐3 trial, found that upfront gastrectomy conferred no survival benefit on patients with advanced gastric cancer with a non‐curable, single‐organ metastasis (e.g., liver, para‐aortic lymph node, peritoneum); in fact, it worsened compliance with chemotherapy and long‐term outcomes, especially in patients with an upper third tumor, most of whom normally undergo a total gastrectomy. These findings suggested that initiating treatment with chemotherapy may be recommended for Stage IV gastric cancer irrespective of the organ with the metastasis. The present study aimed to investigate the clinical course and long‐term outcomes of gastric cancer with oligometastasis to determine the optimal therapeutic strategy.

## 2 MATERIALS AND METHODS

### 2.1 Patients

The patients were retrospectively chosen from an institutional cancer registry and surgical database containing 992 patients who received any form of treatment, such as surgery, chemotherapy, or radiation therapy, for metastatic or recurrent gastric adenocarcinoma at Tokyo Metropolitan Komagome Hospital between 2007 and 2019. Oligometastasis was defined as any of the following: liver metastases (HEP) ≤3; lung metastases (PUL) ≤3; unilateral adrenal gland metastasis (ADR); para‐aortic lymph node metastasis (PALN); or a single‐station, distant, lymph node metastasis, excluding the regional lymph nodes (LYM). Patients with any of the following were excluded: (1) peritoneal dissemination (P1) and/or positivity on peritoneal lavage cytology (CY1); (2) multiple organ metastases; (3) liver metastases >4; (4) lung metastases >4; (5) bone metastasis; or (6) brain metastasis.

### 2.2 Evaluations

The tumors were evaluated using a combination of imaging modalities, such as computed tomography (CT), ultrasonography, magnetic resonance imaging, and radio‐isotope inspection. Diagnostic laparoscopy was performed if peritoneal dissemination was suspected. Clinical tumor depth, nodal status, and pathological response to chemotherapy were assessed using the Japanese Classification of Gastric Carcinoma (JCGC) 15th edition of the Japanese Gastric Cancer Association (JGCA).^14^

### 2.3 Treatment

In the absence of an established treatment strategy for gastric cancer with oligometastasis, the cancer board of Tokyo Metropolitan Komagome Hospital, which consists of gastric surgeons, physicians, oncologists, and pathologists, determined through discussion whether treatment should be started with surgery or chemotherapy and whether chemotherapy should be continued or surgery performed instead based on tumor progression and the patient's physical condition. Patients with metastatic gastric cancer generally received a 5‐fluorouracil (5‐FU)‐ based regimen as the first‐line treatment in accordance with the recommendations of the Japanese gastric cancer treatment guidelines of the JGCA.^8^ If a patient had previously received a 5‐FU‐based regimen or lacked tolerance for it, a taxane or irinotecan (CPT‐11)‐based regimen was administered. Curative surgery was considered for patients who were expected to achieve R0 resection via gastrectomy and/or metastasectomy. If the patient received upfront chemotherapy, surgery was provided to (1) those who were scheduled to receive a few cycles of neoadjuvant chemotherapy and surgery; and (2) those who had a clinical response to chemotherapy (non‐progressive disease, non‐PD) on CT (performed every few months) and were intolerant of the adverse events associated with the chemotherapy. On the other hand, a palliative gastrectomy was provided to patients experiencing symptoms of the primary tumor, such as difficulty with oral food intake or hemorrhaging from tumor, even if the chemotherapy had resulted in PD. The standard lymphadenectomy for advanced gastric cancer was D2. D1 and D3 lymphadenectomy were performed for palliative surgery and para‐aortic lymph node metastasis, respectively. Patients with metachronous oligometastasis with a previous gastrectomy underwent a metastasectomy alone. Postoperative surgical complications were defined as Clavien–Dindo classification grade 2 or higher occurring within 30 postoperative days.^15^ The patients were followed up for at least 5 years with physical examinations, blood tests, and chest‐abdominal CT to assess for tumor progression or recurrence.

### 2.4 Statistical analyses and ethics

Overall survival (OS) was defined as the duration from the initiation of treatment to the patient's death from any cause or the date of the last follow‐up. The Kaplan–Meier method was used to calculate the 5‐year OS and median survival time (MST). The log rank test was performed to compare survival rates by the characteristics of, and treatments for, oligometastasis. Univariate and multivariate Cox proportional hazards models were used to analyze the hazard ratio (HR) for OS. Variables with *p* < 0.1 on univariate analysis were included in multivariate analysis. For all the tests, two‐sided *p* < 0.05 was considered to indicate statistical significance. Statistical analyses were performed using SPSS Statistics, ver. 25 (IBM, Chicago, IL, USA). The present study was approved by the Institutional Review Board of Tokyo Metropolitan Komagome Hospital.

## 3 RESULTS

### 3.1 Patient population

**FIGURE 1:**
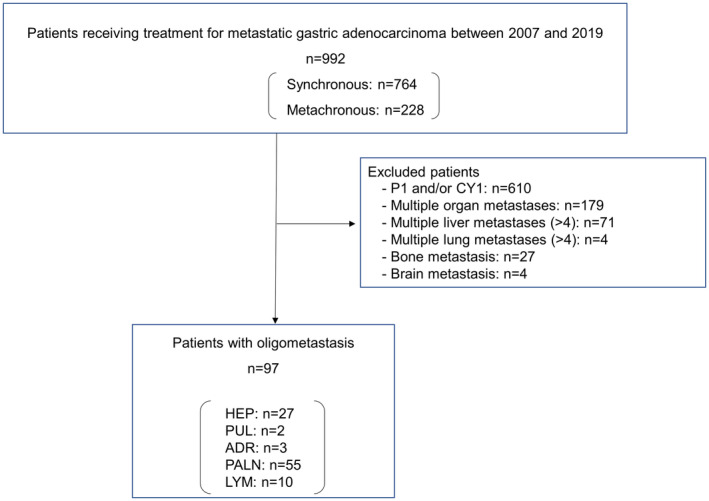
Flow diagram of the patients. ADR, unilateral adrenal gland metastasis; CY1, peritoneal lavage cytology‐positive; HEP, liver metastases ≤3; LYM, single station of distant lymph node metastasis excluding the regional lymph nodes; P1, peritoneal dissemination; PALN, para‐aortic lymph node metastasis; PUL, lung metastases ≤3.

In total, 992 patients received treatment for gastric adenocarcinoma with a distant metastasis at the study center between 2007 and 2019. Figure 1 shows a flow diagram of the patients. Of the total cohort, 764 and 228 patients had synchronous and metachronous metastatic gastric cancer, respectively. The present study enrolled 97 patients (9.8%) after excluding 610 patients with a diagnosis of P1 and/or CY1, 179 patients with multiple organ metastases, 71 patients with liver metastases >4, four patients with lung metastases >4, 27 patients with a bone metastasis, and four patients with a brain metastasis. Table 1 shows the clinical characteristics of the patients. As Table 2 shows, the included patients had the following metastasis sites: HEP (*n* = 27), PUL (*n* = 2), ADR (*n* = 3), PALN (*n* = 55), and LYM (*n* = 10).

**TABLE 1:** Patient characteristics.

	*n*	%
Sex		
Male	77	79.4
Female	20	20.6
Age		
Median (range)	71	49–89
Histological type		
Differentiated	60	61.9
Undifferentiated	37	38.1
Clinical tumor depth		
cT1	3	3.1
cT2	9	9.3
cT3	46	47.4
cT4	39	40.2
Clinical lymph node metastasis		
cN0	10	10.3
cN1	39	40.2
cN2	28	28.9
cN3	20	20.6

**TABLE 2:** Metastasis sites.

	Total		Synchronous		Metachronous	
	*n*	%	*n*	%	*n*	%
HEP	27	27.8	19	25.3	8	36.4
PUL	2	2.1	0	0.0	2	9.1
ADR	3	3.1	1	1.3	2	9.1
PALN	55	56.7	51	68.0	4	18.2
LYM	10	10.3	4	5.3	6	27.3
Total	97		75		22	

Abbreviations: ADR, unilateral adrenal gland metastasis; HEP, liver metastases <3; LYM, metastasis to a single distant lymph node station excluding the regional lymph nodes; PALN, para‐aortic lymph node metastasis; PUL, lung metastases <3.

### 3.2 Treatment

Table 3 shows the patients' treatment details. The majority of the patients were administered chemotherapy as best available regimen, singly or in combination with surgery. Fifty patients received chemotherapy alone, and 47 received surgery with or without chemotherapy. Of the latter, 21, 17, and nine patients underwent a total gastrectomy, distal gastrectomy, and metastasectomy alone, respectively. Postoperative complications more serious than Clavien–Dindo grade 2 occurred in 11 patients (23.4%). The incidence rate of postoperative complications was 23.5%, 33.3%, and 0% in patients who underwent a distal gastrectomy, total gastrectomy, and metastasectomy alone, respectively (*p* = 0.142). R0 resection was done in 42 patients, or 43.3% of the total cohort. Of five patients who underwent R1/2 resection, one was found to have received a postoperative R1 resection for a positive surgical margin. On the other hand, the remaining four patients received the planned R1/2 resection after experiencing symptoms of the primary tumor, such as difficulty with oral food intake or hemorrhaging from the tumor. Of the surgical patients, 21 received preoperative chemotherapy. The median duration of preoperative chemotherapy was 4.2 months (range: 0.7–44.1 months). Nine patients received neoadjuvant chemotherapy and were scheduled for surgery; 10 patients with non‐PD after chemotherapy switched to surgery after experiencing severe adverse effects related to chemotherapy; and two patients with PD after chemotherapy underwent surgery after experiencing the symptoms of the primary tumor. Pathologically complete remission occurred in six patients.

**TABLE 3:** Therapeutic variations and outcomes.

	*n*	%
Treatment		
Surgery alone	3	3.1
Surgery followed by chemotherapy	23	23.7
Chemotherapy followed by surgery	21	21.6
Chemotherapy alone	50	51.5
Initial treatment		
Surgery	26	26.8
Chemotherapy	71	73.2
1st‐line chemotherapy		
5‐FU‐based regimen	83	88.3
S1/CDDP	31	33.0
S1	26	27.7
S1/L‐OHP	10	10.6
S1/CDDP/DTX	4	4.3
FOLFOX	3	3.2
S1/DTX	2	2.1
5FU/LV	2	2.1
Capecitabine/L‐OHP	2	2.1
Capecitabine/CDDP	1	1.1
S1/L‐OHP/DTX	1	1.1
S1/L‐OHP/RAM	1	1.1
5‐FU‐based regimen + trastuzumab	4	4.3
Capecitabine/L‐OHP + trastuzumab	2	2.1
Capecitabine/CDDP + trastuzumab	2	2.1
Non 5‐FU regimen	7	7.4
PTX + RAM	2	2.1
CPT‐11/CDDP	3	3.2
CPT‐11	2	2.1
Type of surgery		
Gastrectomy and metastasectomy	33	70.2
Gastrectomy alone	5	10.6
Metastasectomy alone	9	19.2
Type of gastrectomy		
Total gastrectomy	21	55.3
Distal gastrectomy	17	44.7
Degree of lymphadenectomy		
D1	7	18.4
D2	20	52.6
D3	11	28.9
Postoperative complications (≥CDC‐Grade2)		
Absent	36	76.6
Present	11	23.4
Residual tumor		
R0	42	89.4
R1	1	2.1
R2	4	8.5
Pathological response		
Grade3	6	30.0
Grade2	2	10.0
Grade1b	0	0.0
Grade1a	5	25.0
Grade0	3	15.0
Unknown	4	20.0

Abbreviations: 5‐FU, 5‐fluorouracil; CDC, Clavien–Dindo classification; CDDP, cisplatin; CPT‐11, irinotecan; DTX, docetaxel; FOLFOX, folinic acid/fluorouracil/oxaliplatin; L‐OHP, oxaliplatin; LV, leucovorin; PTX, paclitaxel; RAM, ramucirumab.

### 3.3 Comparison of patient background and short‐term outcomes by the initial treatment

Table 4 shows a comparison of patient background and short‐term outcomes in patients with chemotherapy or surgery as the initial treatment. The clinical nodal status was more progressive in patients with upfront chemotherapy than upfront surgery. The predominant site of oligometastasis was PALN followed by HEP in the upfront chemotherapy group while it was HEP followed by PALN in the upfront surgery group. Upfront chemotherapy did not increase the postoperative complication rate (chemotherapy first: 14.3% vs. surgery first: 30.8%; *p* = 0.164).

**TABLE 4:** Comparison of patient background between initial treatment with chemotherapy and surgery.

	Initiating treatment with chemotherapy (*n* = 71)		Initiating treatment with surgery (*n* = 26)		*p* value
	*n*	%	*n*	%	
Sex					0.779
Male	57	80.3	20	76.9	
Female	14	19.7	6	2.1	
Age					
Median (range)	72	49–88	71	58–89	0.981
Histological type					0.238
Differentiated	41	57.7	19	73.1	
Undifferentiated	30	42.3	7	26.9	
Clinical tumor depth					0.254
cT1	1	1.4	2	7.7	
cT2	8	11.3	1	3.8	
cT3	32	45.1	14	53.8	
cT4	30	42.3	9	34.6	
Clinical lymph node metastasis					0.002
cN0	2	2.8	8	30.8	
cN1	32	45.1	7	26.9	
cN2	21	29.6	7	26.9	
cN3	16	22.5	4	15.4	
Oligometastasis site					0.002
HEP	16	22.5	11	42.3	
PUL	1	1.4	1	3.8	
ADR	0	0.0	3	11.5	
PALN	47	66.2	8	30.8	
LYM	7	9.9	3	11.5	
Oligometastasis timing					0.589
Synchronous	56	78.9	19	73.1	
Metachronous	15	21.1	7	26.9	
Type of surgery					0.203
Total gastrectomy ± metastasectomy	12	57.1	9	34.6	
Distal gastrectomy ± metastasectomy	7	33.3	10	38.5	
Metastasectomy alone	2	9.5	7	26.9	
Postoperative complications (≥CDC‐Grade2)					0.164
Absent	18	85.7	18	69.2	
Present	3	14.3	8	30.8	
Residual tumor					0.786
R0	18	85.7	24	92.3	
R1	1	4.8	0	0.0	
R2	2	9.5	2	7.7	

Abbreviations: ADR, unilateral adrenal gland metastasis; CDC, Clavien–Dindo classification; HEP, liver metastases <3; LYM, metastasis to a single distant lymph node station excluding the regional lymph nodes; PALN, para‐aortic lymph node metastasis; PUL, lung metastases <3.

### 3.4 Survival analyses

**FIGURE 2:**
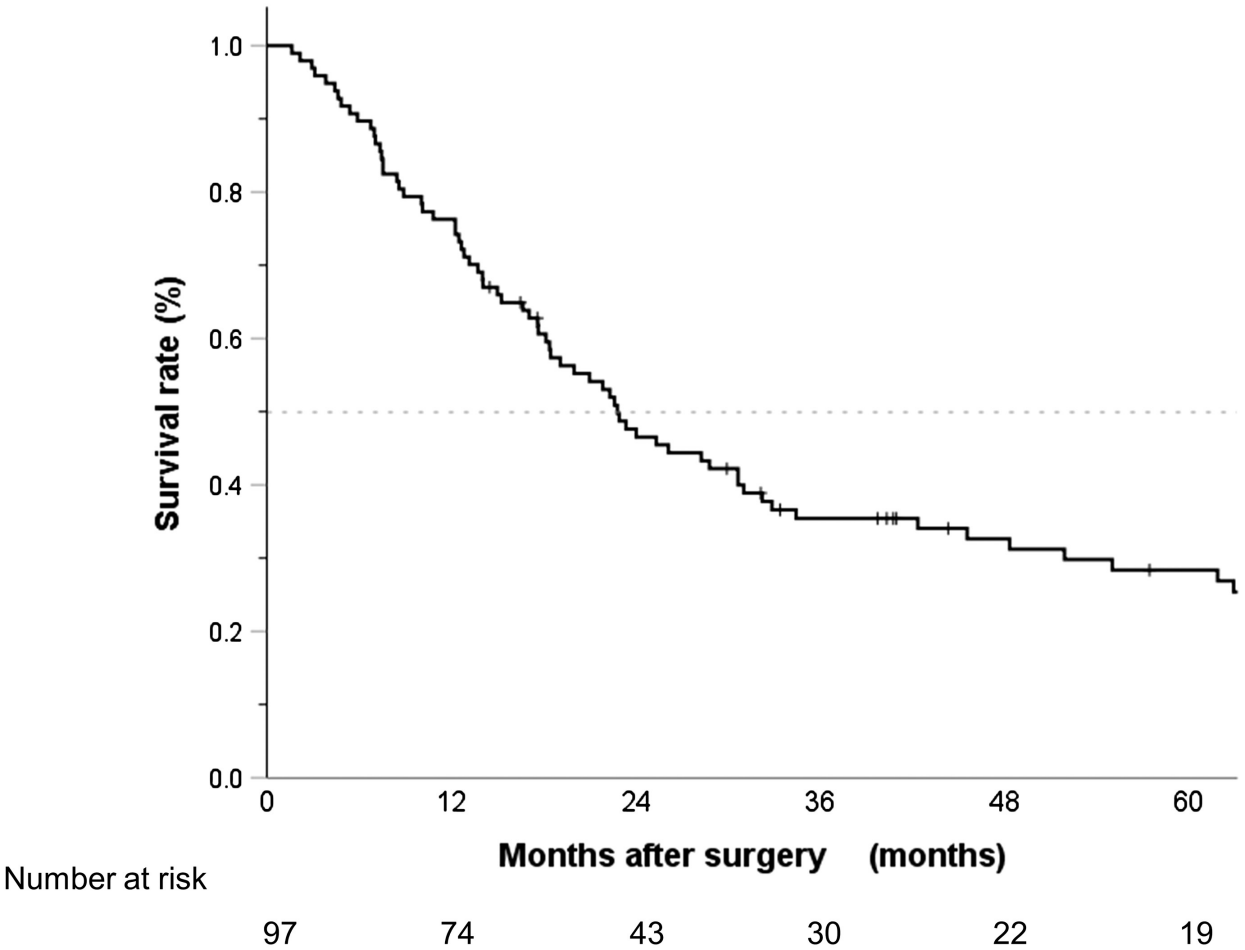
Kaplan–Meier curve of overall survival (OS) in the entire cohort; the median survival time (MST) was 22.8 months (95% confidential interval [CI]: 17.1–28.5), and the 5‐year OS rate was 28.4% (95% CI: 19.3–38.1).

**FIGURE 3:**
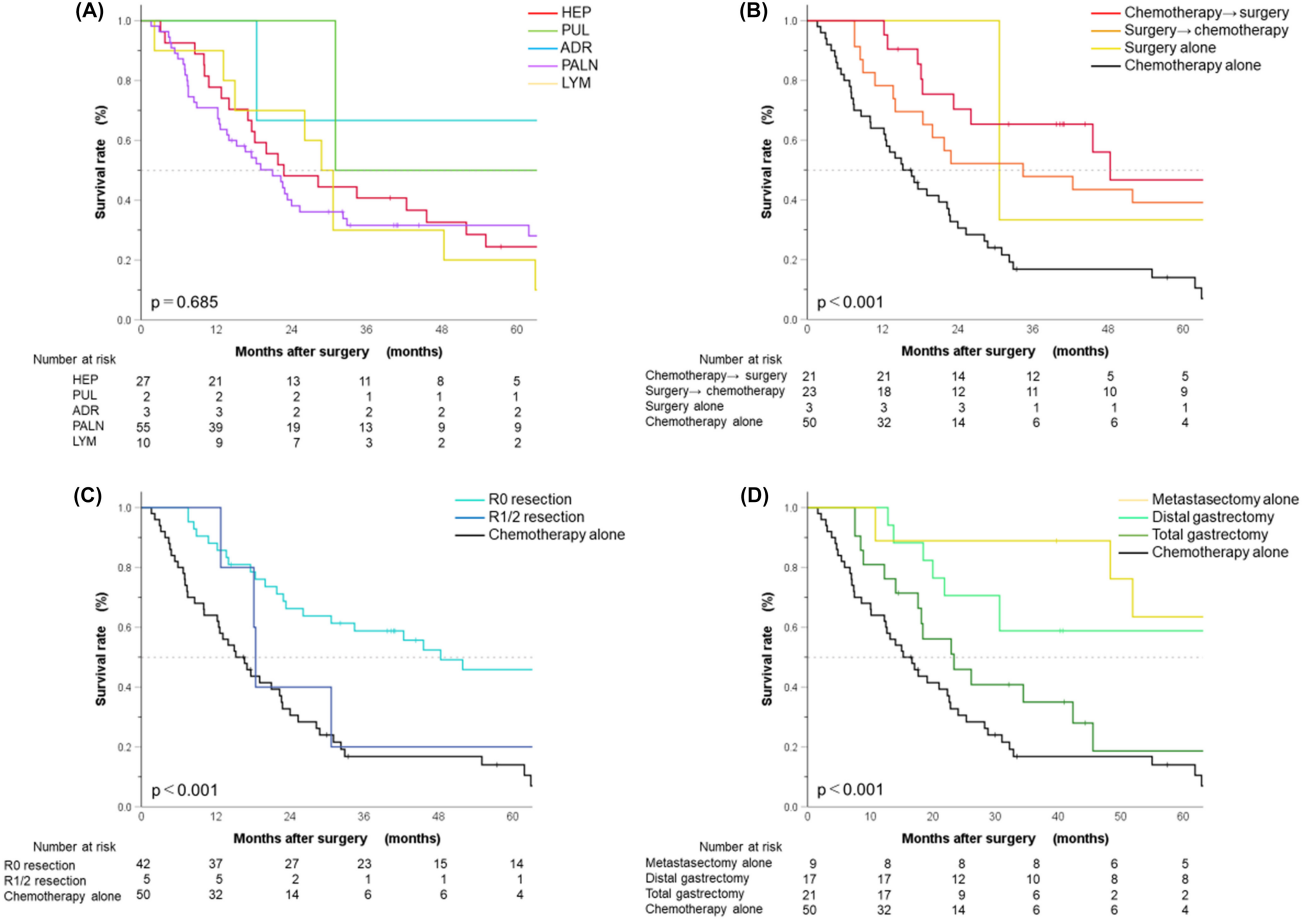
Kaplan–Meier curves of overall survival (OS) in patients stratified by (A) site of metastasis; the respective median survival time (MST) and 5‐year overall survival rate was 28.8 months (95% CI: 8.8–36.8) and 24.4% (95% CI: 10.2–41.9) in patients with HEP, 31.0 months (95% CI: 31.0–not reached) and 50.0% (95% CI: 0.6–91.0) in those with PUL, 82.9 months (95% CI: 0–186.0) and 66.7% (95% CI: 5.4–94.5) in those with ADR, 21.0 months (95% CI: 14.3–27.7) and 31.6% (95% CI: 19.4–44.5) in those with PALN, and 28.8 months (95% CI: 24.1–33.5) and 20.0% (95% CI: 3.1–47.5) in those with LYM (*p* = 0.685), (B) treatment combination; the respective MST and 5‐year OS rate was 48.3 months (95% CI: 17.2–79.5) and 46.7% (95% CI: 20.1–49.6) in patients who received chemotherapy followed by surgery, 34.4 months (95% CI: 2.3–66.5) and 39.1% (95% CI: 19.9–58.0) in those who received surgery followed by chemotherapy, 30.7 months (95% CI: 30.7–not reached) and 33.3% (95% CI: 0.9–77.4) in those who received surgery alone, and 15.3 months (95% CI: 10.2–20.3) and 14.0% (95% CI: 5.7–26.0) in those who received chemotherapy alone (*p* < 0.001), (C) status of residual tumor; the respective MST and 5‐year OS rate was 48.3 months (95% CI: 20.5–76.2) and 45.9% (95% CI: 29.3–60.9) in patients who received R0 resection, and 18.5 months (95% CI: 17.8–19.1) and 20.0% (95% CI: 0.8–58.2) in those who received R1/2 resection (*p* < 0.001), and (D) type of surgery; the respective MST and 5‐year OS rate was 23.4 months (95% CI: 12.3–34.4) and 18.7% (95% CI: 4.0–41.7) in patients who underwent a total gastrectomy, 73.8 months (95% CI: 19.7–not reached) and 58.8% (95% CI: 32.5–77.8) in those with a distal gastrectomy, and 82.9 months (95% CI: 51.0–114.8) and 63.5% (95% CI: 23.8–86.6) in those with a metastasectomy alone (*p* < 0.001).

Figure 2 shows the Kaplan–Meier curve for OS in the total cohort. The MST was 22.8 months (95% CI: 17.1–28.5), and the 5‐year OS rate was 28.4% (95% CI: 19.3–38.1). Figure 3A shows the Kaplan–Meier curve for OS in patients stratified by metastatic site; the results indicated no statistically significant difference (*p* = 0.685). The respective MST and 5‐year OS rate was 28.8 months (95% CI: 8.8–36.8) and 24.4% (95% CI: 10.2–41.9) in patients with HEP; 31.0 months (95% CI: 31.0–not reached) and 50.0% (95% CI: 0.6–91.0) in those with PUL; 82.9 months (95% CI: 0–186.0) and 66.7% (95% CI: 5.4–94.5) in those with ADR; 21.0 months (95% CI: 14.3–27.7) and 31.6% (95% CI: 19.4–44.5) in those with PALN; and 28.8 months (95% CI: 24.1–33.5) and 20.0% (95% CI: 3.1–47.5) in those with LYM. Figure 3B shows the Kaplan–Meier curve for OS in patients stratified by treatment combination; the results indicated a statistically significant difference (*p* < 0.001). The respective MST and 5‐year OS rate was 48.3 months (95% CI: 17.2–79.5) and 46.7% (95% CI: 20.1–49.6) in patients who received chemotherapy followed by surgery; 34.4 months (95% CI: 2.3–66.5) and 39.1% (95% CI: 19.9–58.0) in those who received surgery followed by chemotherapy; 30.7 months (95% CI: 30.7–not reached) and 33.3% (95% CI: 0.9–77.4) in those who received surgery alone; and 15.3 months (95% CI: 10.2–20.3) and 14.0% (95% CI: 5.7–26.0) in those who received chemotherapy alone. The MST and 5‐year OS rate was 45.6 months (95% CI: 23.7–67.5) and 43.7% (95% CI: 27.8–58.6), respectively, in the patients who received a combination of surgery and chemotherapy regardless of the initial therapy (not shown in figure). Figure 3C shows the Kaplan–Meier curve for OS in patients stratified by the status of the residual tumor; the results indicated a statistically significant difference (*p* < 0.001). The respective MST and 5‐year OS rate was 48.3 months (95% CI: 20.5–76.2) and 45.9% (95% CI: 29.3–60.9) in patients who received R0 resection; and 18.5 months (95% CI: 17.8–19.1) and 20.0% (95% CI: 0.8–58.2) in those who received R1/2 resection. Figure 3D shows the Kaplan–Meier curves for OS in patients stratified by surgery type; the results indicated a statistically significant difference (*p* < 0.001). The respective MST and 5‐year OS rate was 23.4 months (95% CI: 12.3–34.4) and 18.7% (95% CI: 4.0–41.7) in patients who underwent a total gastrectomy; 73.8 months (95% CI: 19.7–not reached) and 58.8% (95% CI: 32.5–77.8) in those with a distal gastrectomy; and 82.9 months (95% CI: 51.0–114.8) and 63.5% (95% CI: 23.8–86.6) in those with a metastasectomy alone. Among 47 patients with surgery as well, OS differed significantly between those with, and those without, postoperative complications (5‐year OS rate: 18.2% [95% CI: 2.9–44.2] vs. 51.4% [95% CI: 32.8–67.2]; MST: 18.5 months [95% CI: 4.48–32.5] vs. 65.5 months [95% CI: 35.6–95.3]; *p* = 0.021).

### 3.5 Univariate and multivariate analyses

Table 5 shows the results of univariate and multivariate Cox proportional hazards analyses of the prognostic factors of OS. On multivariate analysis, OS was associated with the age, initial treatment, residual tumor, and surgery type. These factors were entered into the multivariate analysis, which found chemotherapy as the initial treatment (HR: 0.438; 95% CI: 0.193–0.994; *p* = 0.048), distal gastrectomy and/or metastasectomy (HR: 0.290; 95% CI: 0.135–0.322; *p* = 0.001), and R0 resection (HR: 0.373; 95% CI: 0.188–0.740; *p* = 0.005) to be independent, positive factors of OS.

**TABLE 5:** Univariate and multivariate analyses of prognostic factors of overall survival.

	*n*	%	5‐year OS rate (%)	Univariate			Multivariate		
				HR	95% CI	*p* value	HR	95% CI	*p* value
Sex									
Female	77	79.4	37.9	1					
Male	20	20.6	25.6	1.090	0.615–1.931	0.768			
Age (years)									
–75	64	66.0	34.5	1			1		
75–	33	34.0	15.4	1.784	1.100–2.893	0.019	1.613	0.993–2.621	0.053
Clinical tumor depth									
cT1‐3	58	59.8		1					
cT4	39	40.2		1.163	0.721–1.875	0.537			
Histological type									
Differentiated	60	61.9	28.3	1					
Undifferentiated	37	38.1	28.5	1.420	0.883–2.283	0.148			
Oligometastasis timing									
Synchronous	75	77.3	26.4	1					
Metachronous	22	22.7	35.4	0.821	0.476–1.418	0.480			
Oligometastasis site									
HEP	27	27.8	24.4	0.974	0.585–1.623	0.920			
PUL	2	2.1	50.0	0.416	0.058–3.003	0.385			
ADR	3	3.1	66.7	0.458	0.111–1.897	0.281			
PALN	55	56.7	31.6	1.188	0.745–1.893	0.469			
LYM	10	10.3	20.0	1.116	0.553–2.252	0.759			
Type of oligometastasis									
Lymphatic	65	67.0	28.6	1					
Hematogenous	32	33.0	35.5	0.790	0.483–1.292	0.347			
Initial treatment									
Surgery	26	26.8	38.5	1			1		
Chemotherapy	71	73.2	24.2	1.626	0.947–2.790	0.078	0.438	0.193–0.994	0.048
Residual tumor									
Other	55	56.7	14.5	1			1		
R0	42	43.3	45.9	0.353	0.214–0.582	<0.001	0.373	0.188–0.740	0.005
Type of surgery									
Other	71	73.2	15.6	1			1		
DG and/or metastasectomy	26	26.8	60.0	0.261	0.139–0.489	<0.001	0.290	0.135–0.322	0.001

Abbreviations: ADR, unilateral adrenal gland metastasis; CI, confidence interval; DG, distal gastrectomy; HEP, liver metastases <3; HR, hazard ratio; LYM, metastasis to a single distant lymph node station excluding the regional lymph nodes; OS, overall survival; PALN, para‐aortic lymph node metastasis; PUL, lung metastases <3.

## 4 DISCUSSION

The present study aimed to clarify the clinical course and long‐term outcomes in patients with gastric cancer with oligometastasis to determine the optimal therapeutic strategy. Although oligometastasis was rare, comprising only 9.8% of cases of metastatic or recurrent gastric cancer in the present study, the patients with oligometastasis had a favorable prognosis, with a MST of 22.8 months and a 5‐year OS rate of 28.4% in comparison with patients enrolled in recent clinical trials of first‐line chemotherapy for Stage IV gastric cancer, who had a MST of 13–17.5 months and a 2‐year OS rate of less than 20%.^16^, ^17^, ^18^ In particular, the patients in the present study with oligometastasis who received chemotherapy followed by surgery, underwent a distal gastrectomy and/or metastasectomy, and achieved R0 resection showed remarkable, long‐term survival, suggesting that a different therapeutic strategy is needed for patients with oligometastasis deriving from extensively metastatic or recurrent gastric cancer.

Several studies have investigated oligometastatic gastric cancer. A German research team conducted the AIO‐FLOT3 (Arbeitsgemeinschaft Internistische Onkologie–Fluorouracil, Leucovorin, Oxaliplatin and Docetaxel) trial, a prospective, non‐randomized, phase 2 trial enrolling 252 patients with resectable or metastatic gastric or gastroesophageal junction cancer.^10^ They divided the eligible patients into a resectable arm (*n* = 65), limited metastatic arm (*n* = 60), and extensive metastatic arm (*n* = 127). Patients received perioperative chemotherapy with a median of eight (1–15) cycles of FLOT and underwent gastrectomy if restaging showed a chance of R0 resection. Survival analysis of the patients in the limited metastatic arm showed favorable survival, with an MST of 22.9 months in comparison with the patients in the extensive metastatic arm, who had an MST of 10.7 months (HR: 0.37; 95% CI: 0.25–0.55). Although AIO‐FLOT was a small‐scale study and not an RCT, its findings suggested that even metastatic gastric cancer is curable by surgery after chemotherapy if the extent of the metastasis is limited. A large‐scale, nationwide, population‐based study in the Netherlands reported the outcomes of local and/or systemic therapy in 594 patients with esophagogastric cancer with oligometastasis.^9^ Of the total cohort, 83, 22, and 489 patients underwent local treatment alone, both local and systemic therapy, and systemic therapy alone, respectively. The study concluded that local treatment alone (MST: 16.0 months; HR: 0.52; 95% CI: 0.31–0.90) or combination with systemic therapy (MST: 22.7 months; HR: 0.42; 95% CI: 0.22–0.82) improved OS to a greater degree than systemic therapy alone (MST: 8.5 months) in patients with esophagogastric cancer with oligometastasis. While these results had similar tendencies to ours in terms of the benefits of surgery, the MST was lower on the whole than in our study, a discrepancy which may derive from differences in the study population. In particular, it should be noted that the Dutch study included 101 patients (17.0%) with squamous cell carcinoma, with the liver as the chief site of the metastasis (32.3%), followed by extra‐regional lymph nodes (20.9%). In the present study, PALN (56.7%) was the chief site of the metastasis, followed by the liver (27.8%).

Neo‐adjuvant chemotherapy was individually developed for gastric cancer with para‐aortic lymph node (PAN) metastasis in Japan because PAN was originally considered to be a regional lymph node. The JCOG0405, a prospective, multicentric, phase 2 study,^3^ demonstrated a survival benefit of neoadjuvant chemotherapy with S‐1 and cisplatin followed by a gastrectomy and D3 lymphadenectomy in patients with gastric cancer with a bulky, lymph node metastasis and/or para‐aortic lymph node metastasis. This finding was recently highlighted as representative evidence of the efficacy of surgery after chemotherapy for oligometastatic gastric cancer and was incorporated into the recommendations of the latest Japanese gastric cancer treatment guidelines.^8^ This may be one reason accounting for the better OS observed on multivariate analysis in the patients initiating treatment with chemotherapy; the predominant metastatic site was PALN in the latter while it was HEP in the upfront surgery group.

Recently, multicentric studies were conducted in Europe to establish a therapeutic strategy for gastric cancer with oligometastasis. Moreover, the multicentric randomized controlled trial, AIO‐FLOT 5 (the RENAISSANCE trial), which aims to confirm the superiority of peri‐operative chemotherapy with FLOT and surgery to chemotherapy with FLOT alone in patients with oligometastatic adenocarcinoma of the stomach or esophagogastric junction, is currently underway.^19^ In addition, the OligoMetastatic Esophagogastric Cancer (OMEC) project, which consists of five consecutive studies aimed at developing a multidisciplinary, European consensus statement on the definition, diagnosis, and treatment of oligometastatic esophagogastric cancer, has been launched.^20^ The project includes a plan for a prospective, international, multicentric trial as its final stage. The results of these studies will have important implications for this field.

Our study revealed that OS in patients with gastric cancer with oligometastasis was associated with the initiation of treatment with chemotherapy, R0 resection, and distal gastrectomy and/or metastasectomy. The CONVO‐GC‐1,^21^ an international, retrospective cohort study of conversion surgery for Stage IV gastric cancer, also reported that patients with metastatic gastric cancer who underwent R0 resection after systemic chemotherapy were able to improve their long‐term outcomes. The study, enrolling patients who had received chemotherapy and had the opportunity to receive surgery subsequently, found that upfront chemotherapy and R0 resection were essential to improving long‐term outcomes in patients with stage IV gastric cancer.

Compliance with postoperative chemotherapy may explain why the timing of chemotherapy and type of surgery affected long‐term survival. Patients with a gastrectomy reportedly often present with postoperative weight loss associated with poor compliance with postoperative chemotherapy. Long‐term survival in gastric cancer patients and the severity of weight loss were generally worse in patients who received a total gastrectomy than another type of gastrectomy.^22^, ^23^ Moreover, the incidence of postoperative complications may affect the differences in long‐term outcomes among the various types of surgery. Previous studies have reported that postoperative complications impact survival in patients with gastric cancer,^24^, ^25^, ^26^ and the findings of the present study also demonstrated poor OS in patients with postoperative complications. A total gastrectomy involves postoperative complications more frequently than other types of gastrectomy; indeed, the findings of the present study, while statistically non‐significant, bore out this association between total gastrectomy and postoperative complications.

The present study has several limitations. First, it was retrospective and monocentric. Second, the definition of oligometastasis in gastric cancer patients used here is not established. The eligibility criteria in terms of the number of organs involved and the number of metastases per organ differ among studies.^9^, ^10^, ^27^, ^28^ Further discussion of this issue is necessary to arrive at a consensus on the definition of oligometastasis. Third, the study period was extended to more than a decade (2007–2019). The long‐term outcomes of the patients might have been improved during this period by developing a systemic chemotherapy, such as molecularly targeted drugs and immune checkpoint inhibitors,^29^, ^30^, ^31^ which could then have positively influenced the therapeutic strategy for gastric cancer patients with oligometastasis.

In conclusion, although the present study enrolled a small cohort and was retrospective, it was also the first in Japan to investigate gastric cancer with oligometastasis and to demonstrate that long‐term outcomes can be improved if treatment is begun with chemotherapy rather than surgery. Our results warrant a future clinical trial comparing conversion surgery and continuous chemotherapy after achieving a tumor response via chemotherapy.

## AUTHOR CONTRIBUTIONS

Kentaro Hara and Haruhiko Cho made substantial contributions to the study's conception and design. Kentaro Hara, Atsushi Onodera, Kazuya Endo, and Haruhiko Cho made substantial contributions to data acquisition, analysis, and interpretation. Yukio Maezawa, Toru Aoyama, Takanobu Yamada, Takashi Oshima, and Yasushi Rino contributed to drafting the manuscript or revising it critically for important intellectual content. Haruhiko Cho gave final approval of the version to be submitted. Each author has participated sufficiently in the work to be considered an author and agrees to be accountable for all aspects of the work by ensuring that questions related to the accuracy or integrity of any part of the work are appropriately investigated and resolved. All the authors have read and approved the final version of the manuscript.

## FUNDING INFORMATION

This work was not supported by any funding.

## ETHICS STATEMENT

The protocol for this study was approved by the Ethics Committee of the study center (Tokyo Metropolitan Komagome Hospital, Approval No. 2937) and complies with the Declaration of Helsinki and its later amendments. Informed consent was obtained from all the participants.

## CONFLICT OF INTEREST STATEMENT

Takanobu Yamada reports personal fees from Taiho Pharmaceutical Co. Ltd., Bristol‐Myers Squibb Co. Ltd., Johnson and Johnson Co. Ltd., and Ono Pharmaceutical Co. Ltd., outside the submitted work. Yasishi Rino reports personal fees from Bristlo‐Myers Squibb Co. Ltd., Ono Pharmaceutical Co. Ltd., Taiho Pharmaceutical Co. Ltd., Daiichi‐Sankyo Co. Ltd., and Covidien Co. Ltd., outside the submitted work. All remaining authors declare no competing interests.
